# The Anticancer Effect of (1*S*,2*S*,3*E*,7*E*,11*E*)-3,7,11,15-Cembratetraen-17,2-olide(LS-1) through the Activation of TGF-β Signaling in SNU-C5/5-FU, Fluorouracil-Resistant Human Colon Cancer Cells

**DOI:** 10.3390/md13031340

**Published:** 2015-03-16

**Authors:** Eun-Ji Kim, Jung-Il Kang, Jeon-Won Kwak, Chan-Hee Jeon, Nguyen-Huu Tung, Young-Ho Kim, Cheol-Hee Choi, Jin-Won Hyun, Young-Sang Koh, Eun-Sook Yoo, Hee-Kyoung Kang

**Affiliations:** 1 Department of Medicine, School of Medicine, Institute of Medical Sciences, Jeju National University, 102 Jejudaehakno, Jeju 690-756, Korea; E-Mails: ejk8730@naver.com (E.-J.K.); asdkji@hanmail.net (J.-I.K.); jejumed15@naver.com (J.-W.K.); katastrope@naver.com (C.-H.J.); jinwonh@jejunu.ac.kr (J.-W.H.); yskoh7@jejunu.ac.kr (Y.-S.K.); eunsyoo@jejunu.ac.kr (E.-S.Y.); 2 College of Pharmacy, Chungnam National University, Daejeon 305-764, Korea; E-Mails: tunginpc@gmail.com (N.-H.T.); yhk@cnu.ac.kr (Y.-H.K.); 3 Research Center for Resistant Cells and Department of Pharmacology, College of Medicine, Chosun University, Seosuk-dong, Dong-gu, Gwangju 501-759, Korea; E-Mail: chchoi@chosun.ac.kr

**Keywords:** LS-1, SNU-C5/5-FU, apoptosis, TGF-β signaling, carcinoembryonic antigen

## Abstract

The anticancer effect of (1*S*,2*S*,3*E*,7*E*,11*E*)-3,7,11,15-cembratetraen-17,2-olide (LS-1) from *Lobophytum* sp. has been already reported in HT-29 human colorectal cancer cells. In this study, we examined the effect of LS-1 on the apoptosis induction of SNU-C5/5-FU, fluorouracil-resistant human colon cancer cells. Furthermore, we investigated whether the apoptosis-induction effect of LS-1 could arise from the activation of the TGF-β pathway. In SNU-C5/5-FU treated with LS-1 of 7.1 μM (IC_50_), we could observe the various apoptotic characteristics, such as the increase of apoptotic bodies, the increase of the sub-G1 hypodiploid cell population, the decrease of the Bcl-2 level, the increase of procaspase-9 cleavage, the increase of procaspase-3 cleavage and the increase of poly(ADP-ribose) polymerase cleavage. Interestingly, the apoptosis-induction effect of LS-1 was also accompanied by the increase of Smad-3 phosphorylation and the downregulation of c-Myc in SNU-C5/5-FU. LS-1 also increased the nuclear localization of phospho-Smad-3 and Smad-4. We examined whether LS-1 could downregulate the expression of carcinoembryonic antigen (CEA), a direct inhibitor of TGF-β signaling. LS-1 decreased the CEA level, as well as the direct interaction between CEA and TGF-βR1 in the apoptosis-induction condition of SNU-C5/5-FU. To examine whether LS-1 can induce apoptosis via the activation of TGF-β signaling, the SNU-C5/5-FU cells were treated with LS-1 in the presence or absence of SB525334, a TGF-βRI kinase inhibitor. SB525334 inhibited the effect of LS-1 on the apoptosis induction. These findings provide evidence demonstrating that the apoptosis-induction effect of LS-1 results from the activation of the TGF-β pathway via the downregulation of CEA in SNU-C5/5-FU.

## 1. Introduction

Colon cancer is one of the most prevalent cancers in the United States, and incidence rates of colon cancer have been increasing steadily worldwide [1]. There have been remarkable advances in chemotherapy for colon cancer in recent years. Especially, 5-fluorouracil (5-FU), oxaliplatin and irinotecan are often used in combination for the chemotherapy of colon cancer [2,3]. Among them, 5-FU is the anti-metabolite of DNA synthesis by inhibiting thymidylate synthase and was used as the most basic anti-cancer drug in colon cancer and other cancers [4]. However, the increased resistance to anti-cancer drugs is an important factor disturbing cancer treatment. Overcoming drug-resistance is important for the improvement of chemotherapy response and the increase of the survival rate. Interestingly, recent studies indicated that the drug-resistant colon cancer cells could induce a high carcinoembryonic antigen (CEA) level [5]. Moreover, it has been reported that CEA could be upregulated after exposure to 5-fluorouracil in colon and breast cancer cells [6,7].

CEA is a glycosyl phosphatidyl inositol (GPI)-anchored glycoprotein. Normally, CEA is found in both colon and gastrointestinal tissues of a developing fetus in the womb, but the synthesis of CEA stops before birth. Thus, a low level of CEA is maintained in the blood of healthy adults. If the CEA level is raised in the blood of adults, this means that the possibility of developing cancer increases. In cancer patients, an elevated CEA level in blood has also shown poor prognosis and metastasis. Recent studies reported that CEA could contribute to the inhibition of anoikis, a form of apoptosis induced by cell detaching, via interfering with TRAIL-R2 signaling [8] or inactivation of the intrinsic apoptosis pathway [9]. Furthermore, overexpression of CEA has been reported to inhibit apoptosis and transforming growth factor-beta (TGF-β) signaling via CEA directly binding to TGF-β receptor I (TGF-βRI) [10].

**Figure 1:**
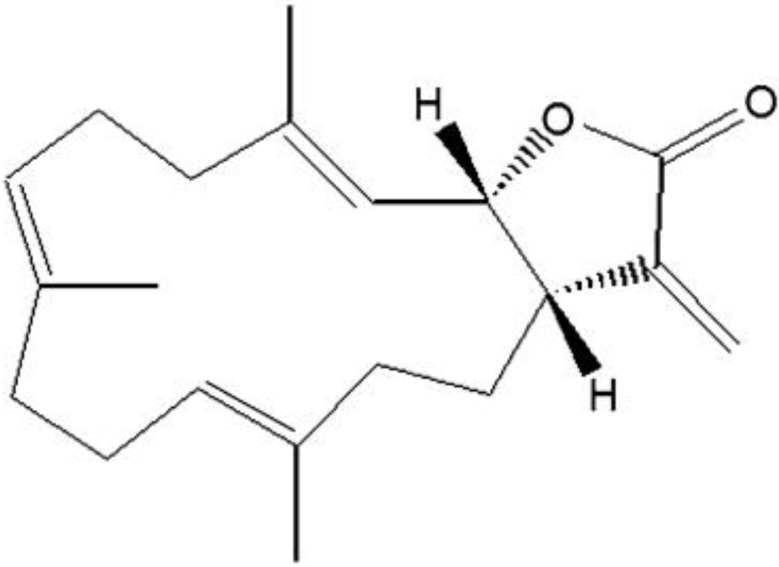
Chemical structure of cembrenolide LS-1.

The TGF-β signaling pathway is involved in many cellular processes, including cell differentiation, apoptosis and other cellular functions [11]. In fact, the TGF-β signaling pathway shows dual roles, such as being a promoter of tumor metastasis and a suppressor of tumor in human cancers [12]. The promotion of tumor metastasis includes the induction of epithelial-mesenchymal transition (EMT), which is improved by TGF-β overexpressed tumor cells at the invasion. The effect of the TGF-β signaling pathway in EMT has been well characterized [13]. Blocking of TGF-β signaling using dominant-negative TGF-βRII prevents mouse skin carcinoma cells from EMT *in vivo* [14]. On the other hand, paradoxically, the activation of the TGF-β signaling pathway has been known to induce tumor suppression [15]. Moreover, the TGF-β signaling pathway is correlated with tumor suppression in the early stages of tumor development [16].

(1*S*,2*S*,3*E*,7*E*,11*E*)-3,7,11,15-Cembratetraen-17,2-olide(LS-1), a marine cembrenolide diterpene, from *Lobophytum sp*. (Figure 1) has been reported to have anticancer effects in HT-29 human colorectal cancer cells via reactive oxygen species (ROS) generation [17,18]. Recent studies reported that overexpression of CEA could inhibit the tumor suppresser effect of the TGF-β signaling pathway via CEA direct interaction with TGF-β receptor I [10]. In the study, we examined the effect of LS-1 on the apoptosis induction of SNU-C5/5-FU, 5-FU-resistant human colon cancer cells. Furthermore, we investigated whether the apoptosis-induction effect of LS-1 could arise from the activation of the TGF-β pathway via the downregulation of CEA.

## 2. Results and Discussion

### 2.1. Results

#### 2.1.1. Effect of LS-1 on the Growth of SNU-C5/5-FU

**Figure 2:**
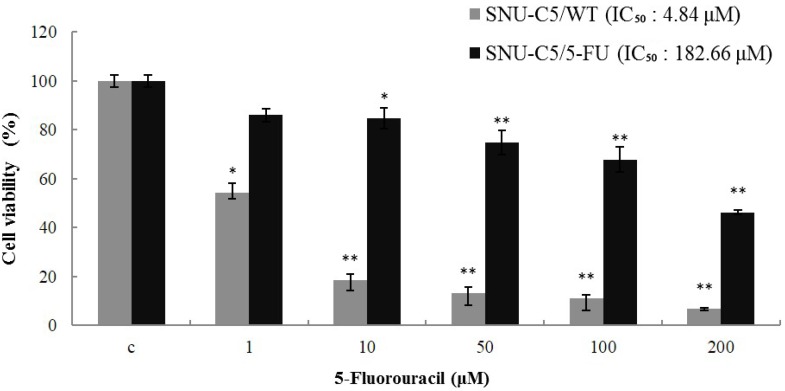
Cytotoxicity of 5-FU in SNU-C5/5-FU and SNU-C5/WT. The cytotoxicity of 5-FU on the cell lines was measured using the methylthiazol tetrazolium (MTT) assay. The data are presented as the mean value ± SD from three independent trials. * *p* < 0.05 and ** *p* < 0.01 compared with the control.

To ascertain whether SNU-C5/5-FU cells have stable resistance to 5-FU, we examined the IC_50_ values for 5-FU in SNU-C5/5-FU and SNU-C5/WT. When 5-FU was treated for 72 h in various concentrations (1, 10, 50, 100 and 200 μM), the IC_50_ (the concentration resulting in 50% inhibition of growth) values of 5-FU in SNU-C5/WT and SNU-C5/5-FU were 4.84 μM and 182.66 μM, respectively (Figure 2). These results indicate that SNU-C5/5-FU is potentially resistant to 5-FU.

**Figure 3:**
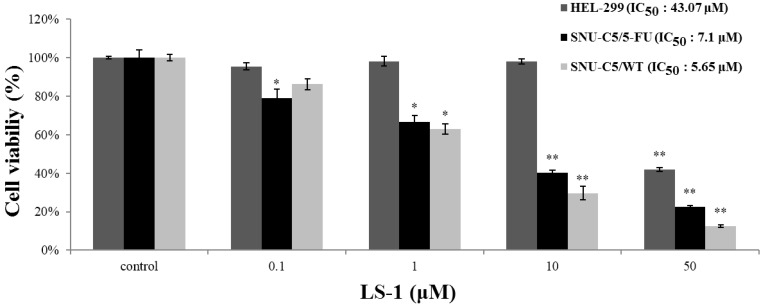
Cytotoxicity of LS-1 in SNU-C5/5-FU, SNU-C5/WT and HEL-299. The cytotoxicity of LS-1 on the cell lines was measured using the MTT assay. The data are presented as the mean value ± SD from three independent trials. * *p* < 0.05 and ** *p* < 0.01 compared with the control.

To evaluate the effect of LS-1 on the proliferation of SNU-C5/5-FU, SNU-C5/WT and HEL-299, a normal fibroblast cell, SNU-C5/5-FU, SNU-C5/WT and HEL-299 were treated with LS-1 (0.1, 1, 10 and 50 μM) for 72 h. Treatment of LS-1 significantly induced cell death of SNU-C5/5-FU and SNU-C5/WT in a dose-dependent manner (IC_50_ = 7.10 and 5.65 μM, respectively), whereas cell death of HEL-299 was scarcely induced even over a 10 μM concentration compared to SNU-C5/5-FU (IC_50_ = 43.07 μM) (Figure 3). The results show that the effect of LS-1 on the induction of cell death affects the cancer cells, including chemotherapeutic agent-resistant cancer cells, such as SNU-C5/5-FU.

#### 2.1.2. Effect of LS-1 on the Apoptosis Induction of SNU-C5/5-FU Cells

Cell death via apoptosis has typical characteristics, such as apoptotic bodies and the increase of sub-G1 hypodiploid cells [19,20]. We thus examined whether the inhibitory effect of LS-1 on the proliferation of SNU-C5/5-FU could result from the induction of apoptosis.

**Figure 4:**
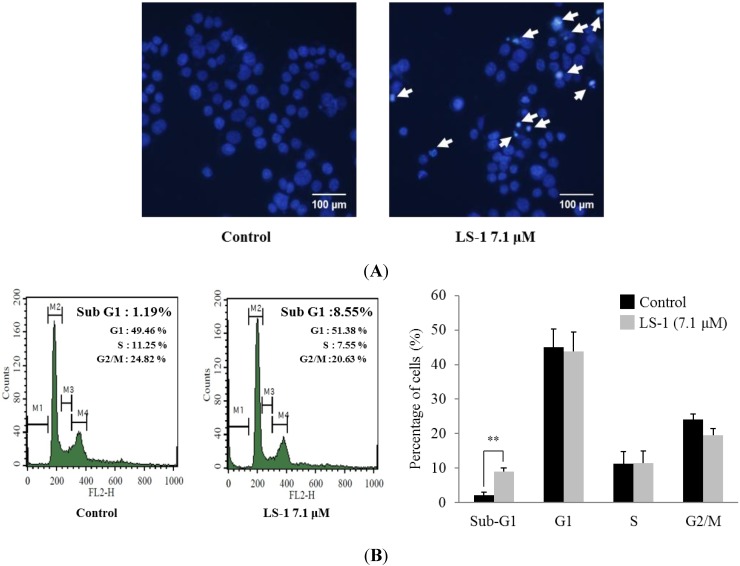

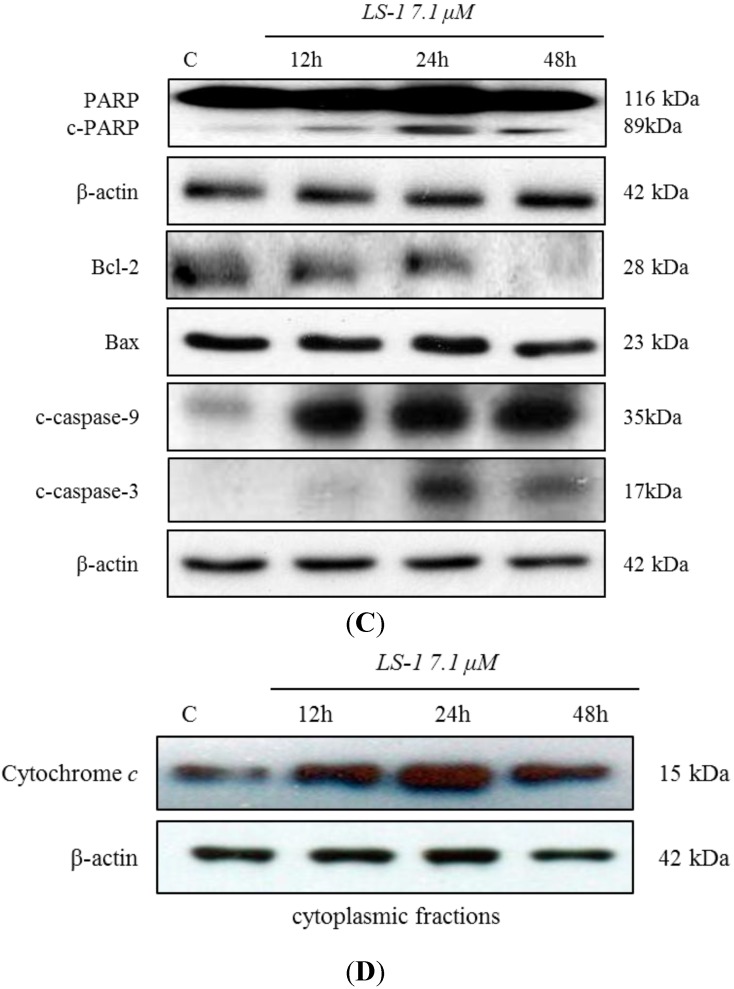
Effect of LS-1 on the induction of apoptosis in SNU-C5/5-FU. (**A**) The SNU-C5/5-FU was treated with LS-1 for 24 h and stained with Hoechst 33,342, which is a DNA-specific fluorescent (10 μg/mL medium at final). Apoptotic bodies were observed in an inverted fluorescent microscope equipped with an IX-71 Olympus camera. (magnification: ×20); (**B**) The SNU-C5/5-FU were treated with LS-1 for 24 h. The cell cycle analysis was performed by flow cytometry. The experiments were performed four times. The data shown are the percentage of cells at that phase of the cell cycle (mean ± SD). ** *p* < 0.01 *versus* control; (**C**) The levels of apoptosis-related proteins were examined by Western blot; (**D**) The levels of cytochrome *c* in the cytoplasmic fractions were examined by Western blot.

When treated with LS-1 of 7.1 μM for 24 h, we could observe the increase of apoptotic bodies (Figure 4A). As shown in Figure 4B, the sub-G1 phase population increased significantly from 1.19% to 8.55% after 24 h of 7.1 μM LS-1 treatment, while the percentages of S and G2/M phase decreased (Figure 4B). Furthermore, treatment with LS-1 regulated the levels of apoptosis-related proteins, such as a decrease of the Bcl-2 level, increase of procaspase-9 cleavage, increase of procaspase-3 cleavage and increase of poly(ADP-ribose) polymerase (PARP) cleavage (Figure 4C). To determine whether LS-1 induced the mitochondrial apoptotic pathway, we measured the effect of LS-1 on the release of cytochrome *c* from mitochondria to the cytosol. As shown in Figure 4D, treatment of LS-1 increased the cytosolic release of cytochrome *c.* These results indicate that LS-1 could inhibit the proliferation of SNU-C5/5-FU via the induction of apoptosis.

#### 2.1.3. Effect of LS-1 on the TGF-β Signaling in SNU-C5/5-FU

**Figure 5:**
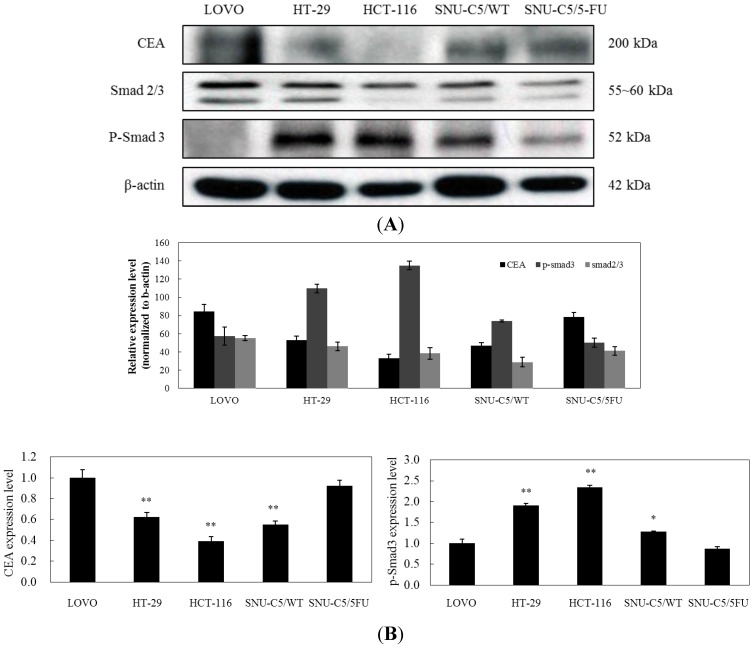
Comparison of CEA and Smad levels in LOVO, HT-29, HCT-116, SNU-C5/WT and SNU-C5/5-FU. (**A**) Levels of CEA and Smad proteins in the cell lines were examined by Western blot. (**B**) Data represent the relative expression percentage of CEA, Smad-2/3 and p-Smad-3 in the cell lines. The data are presented as the mean value ± SD from three independent trials. * *p* < 0.05 and ** *p* < 0.01 compared with the control.

The TGF-β signaling pathway has been known to show the promotion of tumor metastasis or the suppression of tumor, depending on the tumors [12]. On the other hand, recent studies reported that TGF-β could regulate CEA expression [21,22]. Thus, to elucidate the action mechanism of LS-1 on the apoptosis induction of SNU-C5/5-FU, we investigated whether LS-1 could affect the TGF-β signaling in SNU-C5/5-FU. Firstly, we thus examined the characteristics of SNU-C5/5-FU on the TGF-β signaling activation and CEA expression. The activation level of TGF-β signaling was examined as the phosphorylation of Smad-2/3. We also evaluated the CEA level of SNU-C5/5-FU compared with LOVO, CEA high-expressed human colon cancer cells, HT-29, CEA low-expressed human colon cancer cells, HCT-116, CEA none-expressed human colon cancer cells [10], and SNU-C5/WT. The result showed that high-expression of CEA was also accompanied by low activation of TGF-β signaling in LOVO cells, as expected (Figure 5A,B). Relatively speaking, HT-29 and HCT-116 cells showed high activation of TGF-β signaling with low or no CEA expression (Figure 5A,B). Interestingly, SNU-C5/5-FU showed high expression of CEA with low activation of TGF-β signaling compared with SNU-C5/WT (Figure 5A,B). Furthermore, SNU-C5/5-FU and LOVO showed a similar pattern with regard to the expression of CEA, Smad-2/3 and phospho-Smad-3 (Figure 5B). These results suggest that SNU-C5/5-FU has the characteristic of low TGF-β signaling with high-expressed CEA.

**Figure 6:**
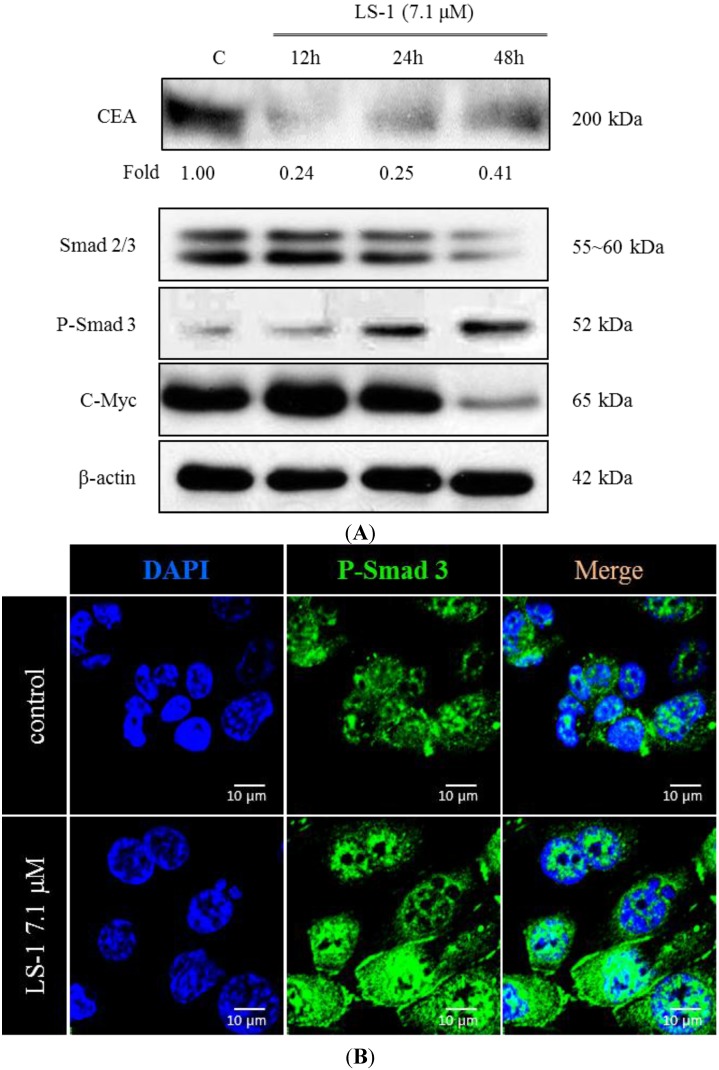

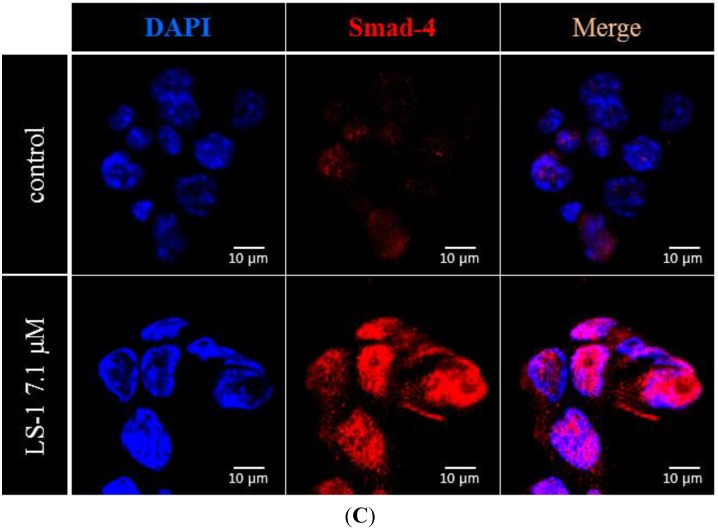
The effect of LS-1 on the expressions of CEA, c-Myc and Smad proteins in SNU-C5/5-FU. (**A**) Modulation of CEA, c-Myc and Smad protein levels by LS-1 was examined by Western blot; (**B**) Modulation of the p-Smad-3 level by LS-1 (7.1 μM, 24 h) was examined by immunofluorescent stain of p-Smad-3; (**C**) Modulation of Smad-4 by LS-1 (7.1 μM, 24 h) was examined by the immunofluorescent stain of Smad-4. The fluorescence was identified via confocal microscopy (FV500, OLYMPUS, New York, NY, USA).

When treated with LS-1 of 7.1 μM, we could observe the increase of Smad-3 phosphorylation and the decrease of c-Myc and CEA, the target proteins of TGF-β signaling (Figure 6A). During activation of TGF-β signaling, phosphorylated Smad-3 combines the Smad-4 and moves into the nucleus [23]. The LS-1 increased the levels of phospho-Smad-3, as well as Smad-4 in the nucleus (Figure 6B,C). These results suggest that LS-1 could induce the activation of TGF-β signaling in SNU-C5/5-FU.

**Figure 7:**
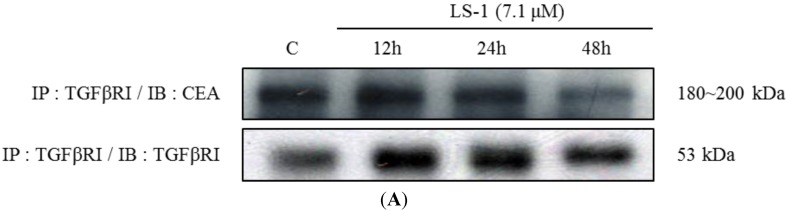

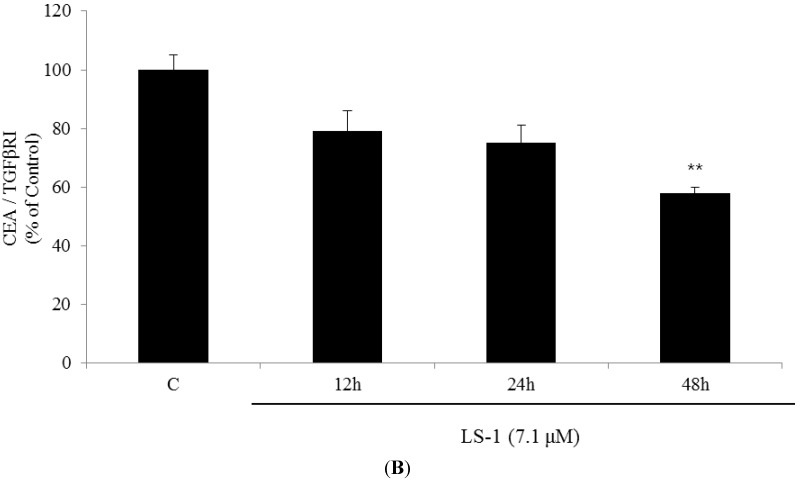
The effect of LS-1 on the interaction between TGFβRI and CEA in SNU-C5/5-FU. (**A**) SNU-C5/5-FU were treated with 7.1 μM of LS-1 for 12, 24 and 48 h. The interaction between TGFβRI and CEA was examined by immunoprecipitation with anti-TGFβRI antibody and with immunoblotting with anti-TGFβRI antibody and anti-CEA antibody; (**B**) Data represent the percentage of CEA expression in SNU-C5/5-FU. The data are presented as the mean value ± SD from three independent trials. * *p* < 0.05 and ** *p* < 0.01 compared with the control.

Recent studies reported that CEA could inhibit TGF-β signaling through CEA direct interaction with TGF-β receptor I (TGFβRI) [10]. We thus examined whether LS-1 could affect direct interaction between TGFβRI and CEA in SNU-C5/5-FU. We observed that TGFβRI could directly interact with the CEA using immunoprecipitation (Figure 7A; control). Furthermore, the amount of CEA combined with TGFβRI was decreased by treatment with LS-1 in a time-dependent manner (Figure 7). The result indicates that LS-1 could inhibit the interaction of CEA and TGFβRI in SNU-C5/5-FU. Consequently, LS-1 seems to effectively activate TGF-β signaling by inhibiting the interaction of CEA and TGFβRI.

#### 2.1.4. LS-1 Induced Apoptosis of SNU-C5/5-FU via Activation of TGF-β Signaling

**Figure 8:**
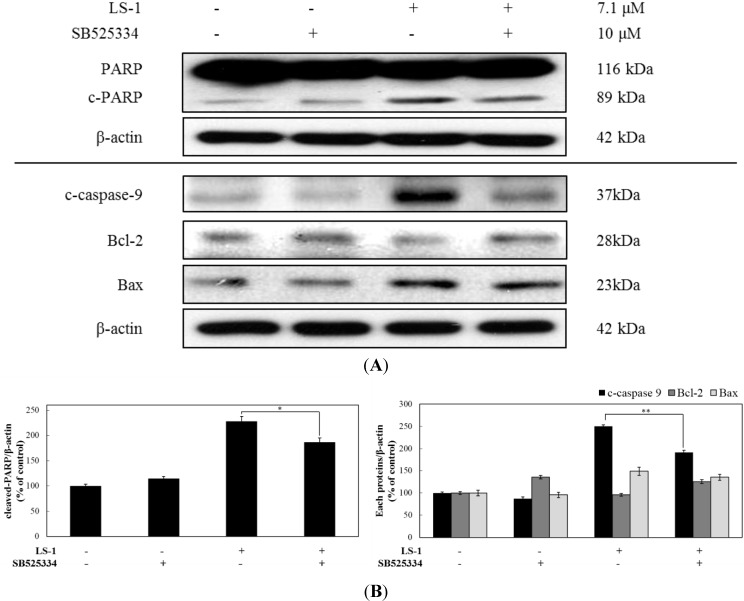
The effect of the TGF-β signaling pathway on the apoptosis-induction effect of LS-1 in SNU-C5/5-FU. (**A**) SNU-C5/5-FU were pre-treated with 10 μM of SB525334 (TGF-βRI kinase inhibitor) and then treated with 7.1 μM of LS-1. The expressions of apoptosis-related proteins were examined by Western blot; (**B**) Data represent the percentage of c-PARP, c-caspase-9, Bcl-2 and Bax expression in SNU-C5/5-FU. The data are presented as the mean value ± SD from three independent trials. * *p* < 0.05 and ** *p* < 0.01 compared with the control.

LS-1 induced apoptosis in SNU-C5/5-FU (Figure 4) and activated TGF-β signaling (Figure 6). In order to examine whether LS-1 may induce apoptosis via activation of TGF-β signaling, we treated with LS-1 and/or SB525334 (TGF-βRI kinase inhibitor). As a result, the blocking of the TGF-β signal by SB525334 inhibited the apoptosis-induction effect of LS-1 in SNU-C5/5-FU (Figure 8). When SNU-C5/5-FU was treated with LS-1 and/or SB525334, SB525334 inhibited the LS-1-induced increase of PARP cleavage and procaspase-9 cleavage, and LS-1-induced downregulation of Bcl-2, while LS-1-induced the increase of Bax, which was not affected by SB525334 (Figure 8A). These results indicated that LS-1 could induce apoptosis via the activation of the TGF-β signaling pathway. Taken together, LS-1 seems to induce apoptosis of SNU-C5/5-FU, which has downregulated the TGF-β pathway with overexpressed CEA, compared to wild-type cells, via the activation of TGF-β signaling with downregulation of CEA.

### 2.2. Discussion

In the study, we examined whether LS-1 could inhibit the proliferation of SNU-C5/5-FU, fluorouracil-resistant human colon cancer cells. To the best of our knowledge, this study is the first to demonstrate that LS-1 could induce the apoptosis of SNU-C5/5-FU via the activation of TGF-β signaling with downregulation of CEA.

LS-1, a marine cembrenolide diterpene, from *Lobophytum sp.* (Figure 1) is reported to have an anticancer effect in HT-29 human colorectal cancer cells [17,18]. Many studies have reported that various marine cembrenolide diterpenes from soft corals, such as *Sinularia gibberosa, Nephthea brassica*, *Lobophytum Crassum*, *Lobophytum* sp., *Sarcophyton glaucum* and *Sarcophyton shrenbergi*, exert cytotoxic effects against several human cancer cells, including HepG2 and MCF-7 cancer cells [24,25,26,27,28,29]. To examine if LS-1 can also inhibit the proliferation of anticancer drug-resistant cancer cells, we used SNU-C5/5-FU cells, which showed a much higher survival rate compared with SNU-C5/WT on 5-FU (Figure 2). Indeed, our results show that LS-1 effectively inhibited the proliferation of SNU-C5/5-FU cells in a dose-dependent manner with an IC_50_ value of 7.1 μM. In addition, LS-1 induced cell death in SNU-C5/WT at lower concentrations than SNU-C5/5-FU (Figure 3). Contrastively, LS-1 barely inhibited the growth of HEL-299, which is a normal cell line, with an IC_50_ value of 43.07 μM (Figure 3). The results show that LS-1 can induce the death of several cancer cells, including SNU-C5/5-FU, fluorouracil-resistant human colon cancer cells.

We know that apoptosis or programmed cell death is the outcome of a complex interplay of pro- and anti-apoptotic molecules. High levels of Bcl-2 expression have been found in several human tumors, and the levels of Bcl-2 expression have been correlated with the aggressiveness of the tumors [30]. Since Bcl-2 functions by forming a heterodimer with its pro-apoptotic partner, such as Bax, the Bcl-2:Bax ratio is proportional to the relative sensitivity or resistance of the cells to various apoptotic stimuli [30]. Apoptotic cell death is induced through two key molecular signaling pathways, the extrinsic and intrinsic pathways. The intrinsic apoptotic pathway is characterized by mitochondrial membrane permeabilization, the release of cytochrome *c* and the formation of apoptosomes. The extrinsic apoptotic pathway is activated in response to ligand binding to death receptors, such as Fas, TNF and TRAIL, and involves the activation of caspase-8. [31]. Here, we showed that LS-1 reduced the Bcl-2 level, whereas the Bax level was rarely changed. In response to LS-1 treatment, caspase-9 was activated, leading to the activation of caspase-3, one of the key executioners of apoptosis. In addition, to clarify the induction of the intrinsic pathway, which works via the activation of caspase-9 by LS-1, we investigated whether LS-1 could affect the release of cytochrome *c* from mitochondria. Treatment of LS-1 increased the cytochrome *c* level in the cytoplasm. When SNU-C5/5-FU cells were treated with LS-1, the activation of caspase-3 was demonstrated by the cleavage of PARP, a nuclear enzyme that is involved in DNA repair in response to various stresses.

TGF-β signaling has important roles in many cellular processes, including apoptosis, cell cycle regulation, cell migration and immune modulation. The TGF-β signaling pathway is initiated by the binding of one of the TGF-β isoforms, which include TGF-β1, TGF-β2 and TGF-β3, on the TGF-β receptor type II (TGF-βRII). The TGF-βRII dimer recruits TGF-βRI and forms the hetero-tetrameric complex. Then, TGF-βRII activates the TGF-βRI via phosphorylation. It induces the recruitment and phosphorylation of receptor-regulated Smad (R-Smad), like Smad-2 and Smad-3. These are substrates for TGF-βRI and act like modulators of the TGF-β signal. Phosphorylated R-Smad could bind with common mediated Smad (Co-Smad) protein, such as Smad-4, followed by complex formation. The phosphorylated R-Smad/Co-Smad complexes translocate into the nucleus, bind transcription promoters and cause the transcription of target gene [23]. Some of the targets of the TGF-β signaling pathway are cell cycle check-point genes, like p15, p21 and p27. Thus, the series of processes evokes G1 arrest in the cell cycle. In cancer, the TGF-β signaling pathway has been known to act as a double-edged sword. By constraining epithelial cell growth, TGF-β performs as a potent tumor suppressor. However, TGF-β also acts as a key player in the induction of EMT, thereby enhancing invasiveness and metastasis. Furthermore, TGF-β signaling has recently been reported to correlate with resistance to anticancer agents [10,32]. Many colorectal cancers escape the tumor-suppressor effects of TGF-β signaling and are resistant to TGF-β-induced growth inhibition [33]. On the other hand, as a tumor marker for colorectal cancers, CEA expression also correlates well with resistance to cytotoxic chemotherapy [34]. TGF-β signaling also contributes to the stimulation of CEA transcription and secretion in colorectal cancer cells [21,22]. Aberrant upregulation of CEA and the alteration of TGF-β signaling are common features of colorectal cancers [23]. CEA has been reported to inhibit apoptosis and the TGF-β signaling pathway through direct interaction with TGFβRI [10]. In the study, we found the downregulation of the TGF-β signaling pathway along with overexpression of CEA in SNU-C5/5-FU compared with SNU-C5/WT (Figure 5). These expression patterns of SNU-C5/5-FU were similar to those of LOVO, another human colon cancer cell line (Figure 5). These results suggest that SNU-C5/5-FU cells might avoid apoptosis by downregulation of the TGF-β signaling pathway along with overexpression of CEA.

The TGF-β signaling pathway modulates the apoptotic pathway, including the death receptor and intracellular signaling pathway [35]. We thus investigated if LS-1 can affect the expression of CEA and the TGF-β signaling pathway in SNU-C5/5-FU. LS-1 treatment decreased the CEA level, while the TGF-β signaling pathway was activated at the concentration inducing apoptosis in SNU-C5/5-FU (Figure 6). Moreover, LS-1 could inhibit the direct interaction between CEA and TGFβRI (Figure 7). These results indicated that LS-1 can activate TGF-β signaling via inhibition of the interactions of CEA and TGFβRI. In sequence, we examined if the effect of LS-1 on the apoptosis induction of SNU-C5/5-FU results from the activation of the TGF-β signaling pathway. When co-treated with LS-1 and SB525334, a selective inhibitor of TGFβRI, we could observe that SB525334 inhibits the apoptosis-induction effect of LS-1 (Figure 8).

Taken together, the results suggest that LS-1 can restore the activity of the TGF-β signaling pathway and induce apoptosis in SNU-C5/5-FU cells. Our studies provide a rationale for the development of LS-1 as a therapeutic agent against human colon cancers, including chemotherapy-resistant colon cancers, especially with the decrease of TGF-β function. On the other hand, TGF-β has been reported to increase intracellular ROS in various cell types [36,37]. ROS induces cell cycle arrest and apoptosis through activating their transcription factors, such as Sp1 [38,39]. In addition, several studies have documented the significant generation of ROS in a variety of cells, which is usually the consequence of mitochondrial respiration and NADPH oxidase (NOX) activity [40,41]. Our previous findings revealed that the anticancer efficacy of LS-1 could be mediated by the induction of apoptosis via ROS generation in human colon cancer cells [18]. In a further study, we will investigate whether LS-1 can generate ROS and induce apoptosis via the activation of TGF-β signaling in SNU-C5/5-FU and the action mechanism of LS-1 on the relationship between the ROS production and the activation of TGF-β signaling.

## 3. Experimental Section

### 3.1. Materials

5-FU, MTT, Hoechst 33342 and propidium iodide (PI) were purchased from Sigma Chemical Co. (St. Louis, MO, USA). Mouse monoclonal anti-c-Myc and anti-smad-4, rabbit polyclonal anti-Bax and anti-TGFβRI and goat polyclonal anti-Smad-2/3 antibodies were purchased from Santa Cruz Biotechnology (Santa Cruz, CA, USA); rabbit monoclonal anti-Bcl-2, anti-p-Smad-3 and anti-cleaved caspase-3, rabbit polyclonal anti-cleaved caspase-9 and anti-PARP and mouse monoclonal anti-CEA antibodies were purchased from Cell Signaling Technology (Beverly, MA, USA); mouse monoclonal β-actin and the selective inhibitor of TGFβRI (SB525334) were purchased from Sigma; Dynabeads^®^ Protein G was purchased from NOVEX^®^ (Invitrogen, Oslo, Norway); aprotinin, leupeptin and Nonidet P-40 were obtained from Roche Applied Science (Indianapolis, IN, USA); the Western blotting reagent, West-zol enhanced chemilumin, was obtained from iNtROn Biotechnology (Gyeonggi, Korea).

### 3.2. Cell Culture

LOVO, HT-29, HCT-116 and SNU-C5/WT, human colon cancer cell lines, were obtained from the Korean Cell Line Bank (KCLB, Seoul, Korea). SNU-C5/5-FU, a human resistant colon cancer cell line, was obtained from the Research Center for Resistant Cells in South Korea. LOVO, HT-29, HCT-116, SNU-C5/WT and SNU-C5/5-FU cells were cultured in RPMI 1640 (Hyclone, UT, USA) medium supplemented with 10% heat-inactivated fetal bovine serum (Hyclone, Logan, UT, USA), 100 U/mL penicillin and 100 mg/mL streptomycin (GIBCO Inc., Grand Island, NY, USA) at 37 °C in a humidified atmosphere with 5% CO_2_. After 2 days, for SNU-C5/5-FU cells, the medium with 140 μM of 5-FU was changed.

### 3.3. Cell Viability Assay

The effect of 5-FU or LS-1 on the growth of SNU-C5/WT, SNU-C5/5-FU and HEL-299 cells was evaluated using the MTT assay [42]. The cells (2 × 10^5^ cells/mL) were seeded on 96-well microplates for 24 h. The cells were treated with 5-FU (1, 10, 50, 100 and 200 μM) or LS-1 (0.1, 1, 10, 20 and 50 μM) for 72 h. After incubation, the cells were treated with 50 μL (5 mg/mL) MTT dye and incubated at 37 °C for 4 h. The medium was aspirated, and 150 μL/well of dimethyl sulfoxide were added to dissolve the formazan precipitate. Cell viabilities were determined by measuring the absorbance at 540 nm using a microplate enzyme-linked immunosorbent assay (ELISA) reader (BioTek Instruments Inc., Winooski, VT, USA). Each experiment was repeated at least three times. Concentration (X-axis)-response (% control optical density; Y-axis) curves were obtained. We determined the IC_50_ values (compound concentration resulting in 50% inhibition of growth).

### 3.4. Morphological Analysis of Apoptosis by Hoechst 33342 Staining

SNU-C5/5-FU cells were seeded at 2 × 10^5^ cells/mL in 1 mL on 24-well microplates. After 24 h of incubation, cells were treated with LS-1 (7.1 μM) for 24 h. The cells were incubated in Hoechst 33342 (Invitrogen, Carlsbad, CA, USA, 10 μg/mL medium at final) at 37 °C for 30 min. SNU-C5/5-FU cells were observed with an inverted fluorescent microscope equipped with an IX-71 Olympus camera (Olympus, New York, NY, USA) and photographed (magnification: ×20).

### 3.5. Flow Cytometric Analysis of Apoptosis

The effect of LS-1 on cell cycle distribution was analyzed by flow cytometry after staining the cells with PI [43]. SNU-C5/5-FU cells (2 × 10^5^ cells/mL) were treated with 7.1 μM of LS-1 for 24 h. The treated cells were detached with trypsin, washed twice with phosphate-buffered saline (PBS) and fixed with 70% ethanol for 30 min at −20 °C. The fixed cells were washed twice with cold PBS, incubated with 50 μg/mL RNase A at 37 °C for 30 min and stained with 50 μg/mL PI at 37 °C for 30 min in the dark. The stained cells were analyzed using fluorescence-activated cell sorter (FACS) caliber flow cytometry (Becton Dickinson, San Jose, CA, USA). Histograms were analyzed with Cell Quest software (Becton-Dickinson). The proportion of cells in the G0/G1, S and G2/M phases was represented as DNA histograms. Apoptotic cells with hypodiploid DNA were measured by quantifying the sub-G1 peak in the cell cycle pattern. For each experiment, 10,000 events per sample were analyzed, and experiments were repeated three times.

### 3.6. Western Blot Analysis

LOVO, HT-29, HCT-116, SNU-C5/WT and SNU-C5/5-FU cells were seeded at 2 × 10^5^ cells/mL. After 24 h, cells were lysed with lysis buffer (50 mM Tris-HCl (pH 7.5), 150 mM NaCl, 2 mM EDTA, 1 mM EGTA, 1 mM NaVO_3_, 10 mM NaF, 1 mM phenylmethylsulfonyl fluoride, 25 μg/mL aprotinin, 25 μg/mL leupeptin, 1 mM Dithiothreitol, 1% Nonidet P-40) for 30 min at 4 °C. To examine the effect of LS-1 in the SNU-C5/5-FU cells, the cells were seeded 2 × 10^5^ cells/mL for 24 h and treated with LS-1 (7.1 μM) for 12, 24 and 48 h. After treatment, SNU-C5/5-FU cells were lysed with lysis buffer for 30 min at 4 °C. The lysates were centrifuged at 15,000 rpm, 4 °C, for 15 min. Protein content was determined according to the method of the Bradford assay [44]. The cell lysates were separated by 6%~15% SDS-PAGE gels and then transferred to polyvinylidene fluoride membrane (BIO-RAD, Hercules, CA, USA) by glycine transfer buffer (192 mM glycine, 25 mM Tris-HCl (pH 8.8) and 20% MeOH (v/v)) at 200 mA for 2 h. After blocking with 5% skim milk solution, the membrane was incubated with primary antibody against PARP (1:2000), cleaved caspase-3 (1:1000), cleaved caspase-9 (1:1000), Bcl-2 (1:1000), Bax (1:1000), CEA (1:1000), Smad-2/3 (1:1000), p-Smad-3 (1:1000), TGFβRI (1:1000), c-Myc (1:1000), cytochrome *c* (1:2000) and β-actin (1:5000) antibodies at 4 °C, overnight, and incubated with a secondary HRP antibody (1:5000; Vector Laboratories, Burlingame, VT, USA) at room temperature for 1 h. Protein bands were detected using a WEST-ZOL^®^ plus Western Blot Detection System (iNtRON, Gyeonggi, Korea) with subsequent exposure to X-ray films (AGFA, Krotich, Belgium).

### 3.7. Co-Immunoprecipitation Assay

SNU-C5/5-FU cells were seeded 2 × 10^5^ cells/mL for 24 h and treated with LS-1 (7.1 μM) for 12, 24 and 48 h. After treatment, SNU-C5/5-FU cells were lysed with lysis buffer for 30 min at 4 °C. The lysates were centrifuged at 15,000 rpm, 4 °C, for 15 min. Dynabeads^®^ Protein G was added directly to mouse monoclonal anti-TGFβRI antibody in 200 μL PBS with 0.02% Tween-20 and incubated with rotation for 10 min at room temperature. The supernatant was then removed. The beads-antibody complex was washed using 200 μL PBS with 0.02% Tween-20. The beads-antibody complex was added directly to the cell lysates and incubated with rotation for 10 min at room temperature. The supernatant was removed, and the beads-antibody-Ag complex was washed using 200 μL PBS with 0.02% Tween-20 3 times. The beads-antibody-Ag complex was mixed with 20 μL of elution buffer (50 mM glycine (pH 2.8)) and 10 μL of NuPAGE lithium dodecyl sulfate (LDS) sample buffer (Invitrogen, Carlsbad, CA, USA) and then heated for 10 min at 70 °C. The supernatant was separated from the beads using a magnet and loaded onto an SDS-PAGE gel.

### 3.8. Confocal Microscopy

SNU-C5/5-FU cells were fixed in 3.5% formaldehyde for 30 min. The fixed cells were permeabilized with 0.1% triton X-100. The cells were blocked in 3% BSA for 1 h at room temperature. The cells were treated with primary mouse monoclonal anti-smad-4 and rabbit monoclonal anti-p-Smad-3 antibodies (1:100) overnight at 4 °C. The immunofluorescence staining of the primary antibodies was performed with Alexa Fluor 488 goat anti-rabbit IgG and Alexa Fluor 594 goat anti-mouse IgG secondary antibody. The fluorescence was identified using confocal microscopy (FV500, OLYMPUS), and the images were acquired at constant photomultiplier tube (PMT), gain, offset, magnification (40 × oil immersion objectives with a zoom factor of 4) and resolution.

### 3.9. Statistical Analyses

Results are shown as means ± standard deviation (SD) from three independent experiments. Student’s *t*-test was used to determine the data with the following significance levels: * *p* < 0.05; ** *p* < 0.01. All assays were performed with at least three independent experiments.

## 4. Conclusions

We previously reported that LS-1 induced apoptosis in HT-29 human colon cancer cells [18]. In conjunction with the report, in this study, our results indicated that LS-1 could induce SNU-C5/5-FU, 5-FU-resistant colon cancer cells, via the activation of the TGF-β pathway with downregulation of CEA. Taken together, our reports suggest that LS-1 may have the potential for use in the treatment of colon cancer, including chemotherapy-resistant colon cancer.
